# Desfechos pós-operatórios com ou sem denosumabe adjuvante em tumores de células gigantes de grau III do fêmur proximal: Um estudo retrospectivo comparativo

**DOI:** 10.1055/s-0045-1809522

**Published:** 2025-07-10

**Authors:** Sheikh Muhammad Ebad Ali, Badaruddin Sahito, Hina Khan, Awais Abro, Sunel Kumar, Muhammad Usman Ali

**Affiliations:** 1 Departamento de Traumatologia e Ortopedia, Chelsea & Westminster Hospital, Londres, Reino Unido; 2 Departamento de Traumatologia e Ortopedia, Dow University of Health Sciences (DUHS), Sindh, Paquistão; 3 Karachi Medical and Dental College (KMDC), Sindh, Paquistão; 4 Ruth KM Pfau Civil Hospital Karachi, Sindh, Paquistão

**Keywords:** denosumabe, fraturas proximais do fêmur, neoplasias ósseas, quadril, tumores de células gigantes, bone neoplasms, denosumab, giant cell tumor, hip, proximal femoral fractures

## Abstract

**Objetivo:**

Comparar retrospectivamente o impacto do uso de denosumabe neoadjuvante em tumores de células gigantes (TCG) de grau III de Campanacci no fêmur proximal com acometimento da articulação do quadril.

**Métodos:**

Avaliamos retrospectivamente 18 casos de TCG de grau III de Campanacci do fêmur proximal que foram submetidos à cirurgia entre janeiro de 2014 e dezembro de 2019 em nosso hospital. Um grupo de 10 pacientes receberam denosumabe neoadjuvante em dose de 120 mg uma vez por semana por 4 semanas, enquanto outro grupo, com 8 pacientes, não recebeu denosumabe antes da cirurgia. Dois pacientes foram submetidos à curetagem intralesional e os demais, à ressecção e artroplastia de quadril. As comparações foram feitas usando o teste *t* não pareado e o teste exato de Fisher. Os resultados funcionais foram avaliados pelo escore revisto da *Musculoskeletal Tumor Society* (MSTS) e pelo escore de quadril de Harris (Harris Hip Score, HSS) em 6 semanas, 6 meses e 12 meses de acompanhamento. A incidência de recidiva também foi determinada.

**Resultados:**

A comparação das médias dos escores MSTS entre os grupos com e sem denosumabe foi de 24,0 ± 6,5 *versus* 20,0 ± 6,0 ( *p* = 0,04) em 6 semanas, respectivamente; 26,0 ± 5,0 *versus* 23,0 ± 0,67 ( *p* = 0,04) em 6 meses, respectivamente; e 28,8 ± 1,7 *versus* 29,5 ± 0,33 ( *p* = 0,35) em 12 meses, respectivamente. A comparação das médias das pontuações no HSS entre os grupos com e sem denosumabe foi de 61,02 ± 7,36 *versus* 48,52 ± 3,97 ( *p* = 0,03) em 6 semanas, respectivamente; 81,1 ± 2,97 *versus* 79,15 ± 3,24 ( *p* = 0,82) em 6 meses, respectivamente; e 89,84 ± 3,75 *versus* 90,05 ± 3,00 ( *p* = 0,38) em 12 meses, respectivamente. Não houve recidiva.

**Conclusão:**

O denosumabe foi clinicamente eficaz na melhora dos desfechos funcionais em curto prazo, mas os desfechos funcionais em longo prazo foram semelhantes entre ambos os grupos. Não observamos aumento na taxa de recidiva no grupo com denosumabe.

## Abstract (en)

**Objective:**

To retrospectively compare the impact of using neoadjuvant denosumab for Campanacci grade-III giant cell tumor (GCT) of the proximal femur involving the hip joint.

**Methods:**

We retrospectively reviewed 18 cases of Campanacci grade-III GCT of the proximal femur receiving surgery between January 2014 and December 2019 from our hospital. One group of 10 patients received weekly neoadjuvant denosumab 120 mg for 4 weeks, while the other group of 8 patients did not receive denosumab before surgery. Two patients were subjected to intralesional curettage while the others received resection and hip arthroplasty. Comparisons were made using unpaired *t* -test and Fisher's exact test. Functional outcomes were assessed by revised Musculoskeletal Tumor Society (MSTS) score and Harris Hip Score (HHS) at 6 weeks, 6 months, and 12 months of follow-up, as well as incidence of recurrence.

**Results:**

The comparison of the mean MSTS scores of the denosumab and non-denosumab groups was as follows: 24.0 ± 6.5 versus 20.0 ± 6.0 ( *p* = 0.04) at 6 weeks respectively; 26.0 ± 5.0 versus 23.0 ± 0.67 ( *p* = 0.04) at 6 months respectively; and 28.8 ± 1.7 versus 29.5 ± 0.33 ( *p* = 0.35) at 12 months respectively. The comparison of HHSs between the denosumab and non-denosumab groups was as follows: 61.02 ± 7.36 versus 48.52 ± 3.97 ( *p* = 0.03) at 6 weeks respectively; 81.1 ± 2.97 versus 79.15 ± 3.24 ( *p* = 0.82) at 6 months respectively; and 89.84 ± 3.75 versus 90.05 ± 3.00 ( *p* = 0.38) at 12 months respectively. There was no recurrence.

**Conclusion:**

Denosumab was clinically effective in improving the short-term functional outcomes, but long-term functional outcomes remained similar between the groups. We did not find an increased recurrence rate in the denosumab group.

## Introdução

O tumor de células gigantes (TCG) é um tumor benigno, mas localmente agressivo, da extremidade de um osso longo. É caracterizado pelo crescimento de células estromais mononucleares e pela presença de muitas células gigantes multinucleadas em distribuição homogênea. 1 O fêmur distal, a tíbia proximal e o rádio distal são os sítios mais frequentes de TCGs na região metafisária-epifisária. 1 O TCG do fêmur proximal é uma doença rara, com maior morbidade devido a distúrbios na marcha, alterações de estilo de vida, risco de fraturas patológicas e limitações nas atividades diárias. Além disso, a doença ocorre em uma população mais jovem, com maior risco a ter uma longa qualidade de vida ruim. Portanto, o TCG do fêmur proximal merece atenção especial. O TCG tem uma ligeira prevalência no sexo feminino e é mais frequente entre a terceira e a quarta décadas de vida. 2 No sistema de classificação de Campanacci, o grau III é atribuído ao TCG com margens mal definidas, perda óssea cortical e invasão dos tecidos moles circundantes. 3

O denosumabe é o único medicamento aprovado pela Food and Drug Administration (FDA) dos Estados Unidos para o tratamento de TCG. 4 O denosumabe diminui o número de células gigantes multinucleadas na matriz óssea e acelera o desenvolvimento da borda madura ao redor do tumor ao bloquear o ativador do receptor da via do fator nuclear-κB (RANK)/RANK-ligante (RANKL). O denosumabe foi empregado como um método de terapia neoadjuvante em estudos anteriores que, entretanto, tiveram um número substancialmente maior de ciclos do medicamento no período pré-operatório. 5

Este foi o primeiro estudo que visou investigar os resultados funcionais e oncológicos do denosumabe neoadjuvante no TCG de grau III de Campanacci do fêmur proximal em associação à cirurgia. Os estudos anteriores sobre TCG do fêmur proximal focaram principalmente na ressecção e artroplastia do fêmur proximal com endoprótese, prótese de Wagner, artroplastia total do quadril e hemiartroplastia do quadril. 6 7 Esses estudos não consideraram a curetagem intralesional como opção, exceto por Wijsbek et al., que realizaram este procedimento em 10 pacientes, mas não usaram denosumabe neoadjuvante. 8 Considerando a idade média da população, uma abordagem de resgate articular seria mais apropriada do que o uso de próteses, que têm duração limitada e, após certo tempo, podem sofrer afrouxamento. 9 Todos os estudos realizados até o momento avaliaram os resultados oncológicos pelo escore da Musculoskeletal Tumor Society (MSTS) sem utilizar outras pontuações de quadril para avaliar o desfecho funcional e a qualidade de vida após a cirurgia.

## Materiais e Métodos

### Delineamento do Estudo

Os dados deste estudo foram adquiridos retrospectivamente dos arquivos de prontuários dos pacientes tratados entre janeiro de 2014 e dezembro de 2019 em um hospital terciário. Os dados dos pacientes incluíram nome, idade, diagnóstico com radiografias e laudos de biópsia, osso acometido, plano de tratamento, estado no último acompanhamento e contato para correspondência. Os pacientes foram contatados individualmente pelo segundo autor para assinatura do termo de consentimento livre e esclarecido antes do registro no estudo, que seguiu a Declaração de Helsinque. O trabalho recebeu a aprovação de nosso Comitê de Ética sob número CHK/DMC/Ortho/Dated 11–22/001.

### Critérios de Inclusão e Exclusão

Os pacientes com diagnóstico de TCG de grau III de Campanacci do fêmur proximal confirmado por imagem e submetidos à cirurgia foram incluídos no estudo. O TCG do fêmur proximal incluiu o TCG de marcos anatômicos da cabeça, colo e áreas trocantéricas e subtrocantéricas. A população do estudo foi estratificada em duas coortes de acordo com a administração de injeções subcutâneas pré-operatórias de denosumabe em dose de 120 mg, uma vez por semana, durante 4 semanas. Todos os pacientes submetidos à cirurgia, mas que não receberam denosumabe, foram estratificados no grupo sem denosumabe, enquanto aqueles que foram submetidos à cirurgia e que receberam denosumabe foram incluídos no grupo com denosumabe.

A exclusão foi feita com base na imaturidade esquelética e ausência de idade nos prontuários. Pacientes com TCG maligno primário ou secundário, TCG de grau I ou II, acompanhamento por menos de 12 meses, não conformidade com a reabilitação pós-operatória, que receberam menos de 4 doses de denosumabe, bem como aqueles submetidos à curetagem prévia do TCG ou que não deram seu consentimento também foram excluídos do estudo.

### Técnica Cirúrgica

A escolha de um dos dois métodos a seguir foi feita após discussão na reunião pré-operatória da equipe multidisciplinar do conselho de tumores, que reavaliou os TCGs após quatro doses de denosumabe.

- Curetagem intralesional com ressecção hemicortical.
- Ressecção e artroplastia de quadril.

#### Curetagem intralesional com ressecção hemicortical

**Fig. 1:**
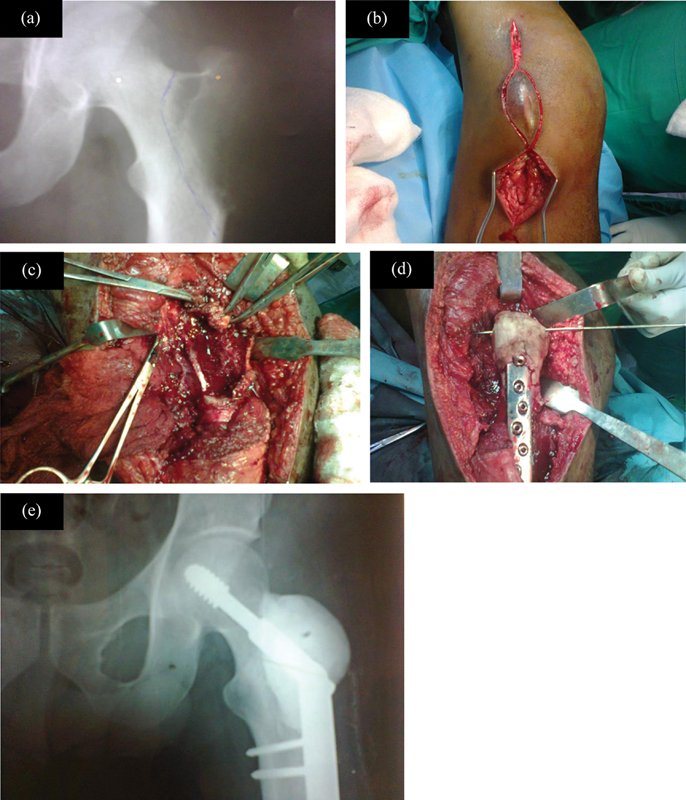
Métodos cirúrgicos. ( **A** ) Radiografia pré-operatória após terapia com denosumabe. ( **B** ) Incisão em formato de barco. ( **C** ) Após a criação de janela cortical e a curetagem em alta velocidade. ( **D** ) Preenchimento com aloenxerto ósseo, cimento e parafuso dinâmico de quadril (DHS). ( **E** ) A radiografia pós-operatória mostra o DHS e o aloenxerto com cimento ósseo.

Após consentimento e aconselhamento e sob medidas assépticas, por meio de abordagem lateral, a pele, o tecido subcutâneo e a fáscia foram incisados e o tumor foi localizado. Sob o braço C, uma janela cortical foi feita por ressecção das margens, incluindo a parede lateral do colo e o trocanter maior. O tumor residual na parede medial foi curetado e a broca foi usada para remoção do tumor restante e nivelamento da superfície. Com a superfície lisa e livre de tumor, cauterização e peróxido de hidrogênio foram usados para queima de micropartículas do tumor. Um parafuso de quadril dinâmico foi colocado e o defeito foi reconstruído com cimento ósseo. Os orifícios foram criados no cimento ósseo e os abdutores foram reinseridos. A Fig. 1 mostra as técnicas cirúrgicas.

Depois da lavagem final, a ferida foi fechada em planos. O movimento do quadril, assim que possível, foi encorajado no pós-operatório, e a sustentação de peso foi retomada assim que os pacientes conseguissem tolerá-la. Os pontos foram removidos após 2 semanas e os pacientes foram acompanhados a cada 15 dias por 3 meses, depois uma vez ao mês por 6 meses, a cada 3 meses por 2 anos e, então, duas vezes ao ano. Em cada consulta, os movimentos do quadril foram avaliados e documentados em gráficos de acompanhamento.

#### Ressecção e Artroplastia de Quadril

Sob anestesia geral, com consentimento do paciente e medidas antissépticas, uma incisão cutânea lateral longitudinal em posição lateral foi feita após a avaliação das radiografias. Após a incisão pelo tecido subcutâneo e fáscia, os músculos foram refletidos. Depois, as medidas foram obtidas e o fêmur foi seccionado a 2 a 3 cm da margem do tumor. A ressecção de margem ampla do TCG foi realizada e o espécime foi obtido para histopatologia das margens. Após a ressecção, o sítio foi avaliado quanto à adequação da prótese de Wagner.

- A hemiartroplastia bipolar foi realizada em pacientes com fratura patológica do colo do fêmur ou lesão do calcar femoral.
- Utilizamos a prótese de Wagner com cabeça bipolar em tumores que se estendiam para a área intertrocantérica ou subtrocantérica sem acometimento da cartilagem do acetábulo.
- A artroplastia total do quadril com prótese de Wagner foi usada em pacientes com lesões na cartilagem e/ou no calcar femoral.

**Fig. 2:**
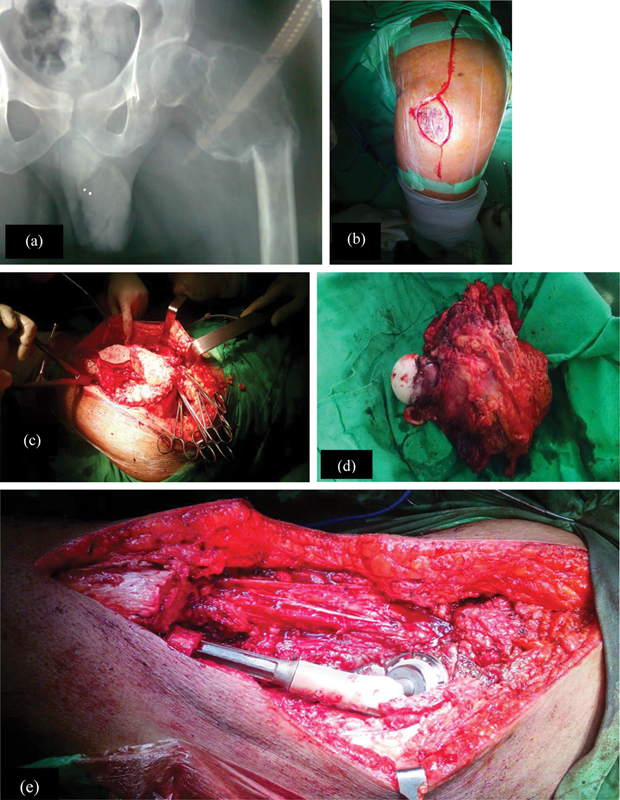
Métodos cirúrgicos. ( **A** ) Radiografia pré-operatória após terapia com denosumabe. ( **B** ) Incisão em formato de barco. ( **C** ) Incisão de pele, tecido subcutâneo e fáscia para exposição do tumor. ( **D** ) Espécime excisado do fêmur proximal. ( **E** ) Inserção de prótese de Wagner e artroplastia total do quadril.

O sítio cirúrgico foi lavado e a ferida fechada em planos. Curativos estéreis foram aplicados em dias alternados e as suturas foram retiradas após 2 semanas. Os pacientes foram acompanhados a cada 15 dias por 3 meses, depois uma vez ao mês por 6 meses, a cada 3 meses por 2 anos e, então, duas vezes ao ano. Em cada consulta, os movimentos do quadril foram avaliados e documentados em gráficos de acompanhamento. A Fig. 2 mostra as técnicas cirúrgicas.

### Análise Comparativa de Desfechos

Nosso resultado primário foi composto pelos desfechos pós-operatórios da articulação do quadril, avaliados em 6 semanas, 6 meses e 12 meses de acompanhamento pelo escore de quadril de Harris (Harris Hip Score, HSS) e o escore revisto da MSTS. De acordo com o HSS, de 0 a 25% indica resultado ruim, de 26 a 50%, resultado razoável, de 51 a 75%, resultado bom e de 76 a 100%, resultado excelente, e de acordo com o escore revisto da MSTS para o membro inferior, de 0 a 7 significa resultado ruim, de 8 a 14, resultado razoável, de 15 a 22, resultado bom e, acima de 22, resultado excelente, após 6 semanas, 6 meses e 12 meses de acompanhamento; o resultado secundário foi a incidência de recidiva.

### Análise Estatística

Todas as estatísticas descritivas são representadas como médias e desvios-padrão para variáveis contínuas. Variáveis categóricas são mostradas como frequências e porcentagens. As características basais dos dois grupos foram comparadas pelo teste *t* não pareado (variáveis contínuas) ou pelo teste exato de Fisher para proporções de duas variáveis categóricas, que é mais adequado para estudos com amostra de menor tamanho e IC de 95%. Os dados foram analisados usando o software IBM SPSS Statistics for Windows (IBM Corporation), versão 22.0. O HSS e o escore MSTS são variáveis contínuas, enquanto a incidência de recidiva e complicações são variáveis categóricas.

## Resultados

**Fig. 3:**
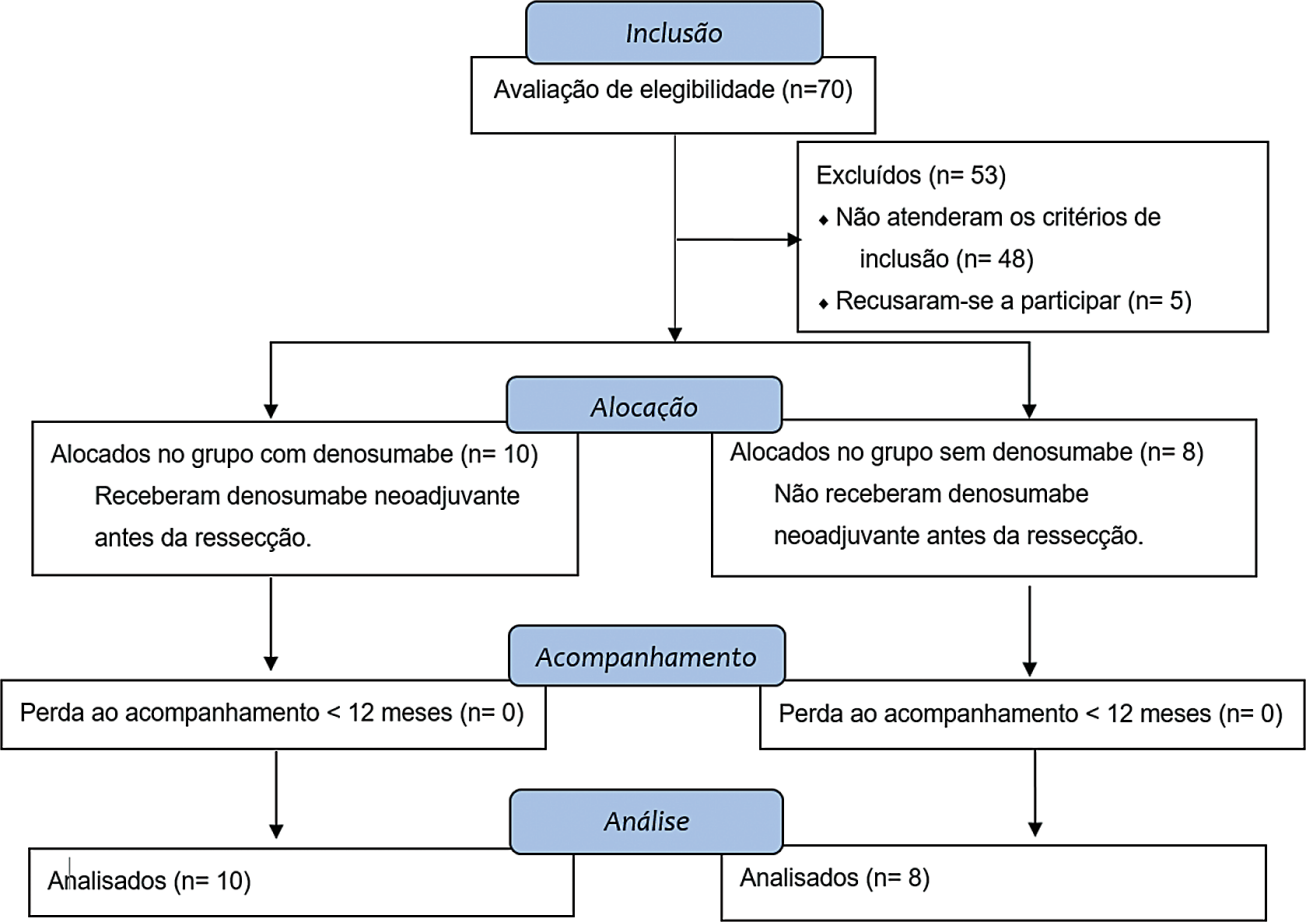
Fluxograma de inclusão/exclusão de pacientes.

Nenhuma das características do estudo diferiu significativamente entre os grupos com e sem denosumabe, incluindo a idade média (28,6 ± 5,85 *versus* 29,5 ± 2,96, respectivamente; *p* = 0,81), sexo (4:6 [40%:60%] *versus* 0:8 [0%:100%], respectivamente; *p* = 0,09), fraturas patológicas (8 [80%] *versus* 4 [50%], respectivamente; *p* = 0,32), recidiva do TCG (2 [20%] *versus* 0 [0%], respectivamente; *p* = 0,47) ou acompanhamento médio em meses (21,62 ± 5,13 *versus* 26,82 ± 9,48, respectivamente; *p* = 0,38). As partes acometidas do fêmur proximal nos grupos com e sem denosumabe são cabeça (0 [0%] *versus* 0 [0%], respectivamente; *p* = 1), colo (2 [20%] *versus* 2 [25%], respectivamente; *p* = 1), área intertrocantérica (4 [40%] *versus* 2 [25%], respectivamente; *p* = 0,64) e área subtrocantérica (4 [40%] *versus* 4 [50%], respectivamente; *p* = 1). Todos os pacientes no grupo sem denosumabe foram submetidos à ressecção e artroplastia. No grupo com denosumabe, 2 (20%) pacientes foram submetidos à curetagem e os demais à ressecção e artroplastia. A curetagem intralesional (20% versus 0%; *p* = 0,48) e a ressecção e artroplastia (80% versus 100%; *p* = 0,48) foram estatisticamente semelhantes entre os grupos com e sem denosumabe, respectivamente. Os resultados são mostrados na Tabela 1 , e os critérios de inclusão e exclusão de pacientes são mostrados em um fluxograma na Fig. 3 .

**Tabela 1:** Características basais para comparação entre os grupos com e sem denosumabe

	Com denosumabe	Sem denosumabe	Valor de *p*
Número de pacientes	10	8	−
Idade em anos: média ± desvio padrão	28,6 ± 5,85	29,5 ± 2,96	0,81
Sexo: n (%)			
Masculino	4 (40%)	0 (0%)	0,09
Feminino	6 (60%)	8 (100%)	
Fraturas patológicas: n (%)	8 (80%)	4 (50%)	0,32
Acompanhamento (meses): média ± desvio padrão	21,62 ± 5,13)	26,82 ± 9,48)	0,38
Procedimento cirúrgico: n (%)			
Curetagem intralesional com ressecção hemicortical	2 (20%)	0 (0%)	0,48
Ressecção e artroplastia de quadril	8 (80%)	8 (100%)	
Recidiva de tumor de células gigantes: n (%)	2 (20%)	0 (0%)	0,47
Parte acometida do fêmur proximal: n (%)			
Cabeça	0 (0%)	0 (0%)	1
Colo	2 (20%)	2 (25%)	1
Intertrocantérica	4 (40%)	2 (25%)	0,64
Subtrocantérica	4 (40%)	4 (50%)	1

### Escore MSTS

Os escores médios no MSTS nos grupos com e sem denosumabe às 6 semanas (24 ± 6,5 *versus* 20,0 ± 6,0, respectivamente; *p* = 0,04) e aos 6 meses (26,0 ± 5 *versus* 23,0 ± 0,67, respectivamente; *p* = 0,04) após a cirurgia apresentaram diferenças estatisticamente significativas. Às 6 semanas, os escores MSTS foram bons no grupo sem denosumabe e excelentes no grupo com denosumabe. Os escores MSTS foram excelentes nos 2 grupos a partir dos 6 meses. No entanto, observamos melhora no alívio da dor e nas emoções que se estenderam da fase pós-operatória imediata até 6 meses de acompanhamento no grupo tratado com denosumabe. O escore médio de dor foi de 3,9 ± 0,4 no grupo com denosumabe versus 2,1 ± 0,6 no grupo sem denosumabe. Entretanto, o escore médio no MSTS foi semelhante entre os grupos com e sem denosumabe no acompanhamento de 12 meses (28,8 ± 1,7 *versus* 29,5 ± 0,33, respectivamente; *p* = 0,35). Esses achados são resumidos na Tabela 2 .

**Tabela 2:** Comparação de desfechos pós-operatórios entre os grupos com e sem denosumabe

	Com denosumabe	Sem denosumabe	Valor de *p*
Escore MSTS: média ± desvio padrão			
6 semanas	24,0 ± 6,5	20,0 ± 6,0	0,04
6 meses	26,0 ± 5,0	23,0 ± 0,67	0,04
12 meses	28,8 ± 1,7	29,5 ± 0,33	0,35
HSS: média ± desvio padrão			
6 semanas	61,02 ± 7,36	48,52 ± 3,97	0,03
6 meses	81,1 ± 2,97	79,15 ± 3,24	0,82
12 meses	89,84 ± 3,75	90,05 ± 3,00	0,38
Recidiva: n (%)	0 (0%)	0 (0%)	1
Infecções	0 (0%)	1 (12,5%)	0,44
Luxação de quadril	0 (0%)	1 (12,5%)	0,44

**Abreviaturas:** HSS, Harris Hip Score (Escore de Quadril de Harris); MSTS, Musculoskeletal Tumor Society.

### Escore de Quadril de Harris

A pontuação média no HSS continuou excelente e sem diferenças estatisticamente significativas entre os grupos com e sem denosumabe aos 6 meses (81,1 ± 2,97 *versus* 79,15 ± 3,24, respectivamente; *p* = 0,82) e 12 meses (89,84 ± 3,75 *versus* 90,05 ± 3,00, respectivamente; *p* = 0,38) de acompanhamento pós-operatório. No entanto, os resultados foram consideravelmente contrastantes entre os grupos com e sem denosumabe às 6 semanas de acompanhamento (61,02 ± 7,36 *versus* 48,52 ± 3,97, respectivamente; *p* = 0,03). Às 6 semanas de acompanhamento, os resultados foram bons no grupo com denosumabe e razoáveis no grupo sem denosumabe. Considerando a escala de dor do HSS, a dor foi significativamente maior no grupo sem denosumabe do que no grupo com denosumabe nos primeiros 6 meses, o que pode ser atribuído à maior disfunção do quadril no grupo sem o tratamento. Entretanto, a diferença na dor tornou-se não significativa após 12 meses de acompanhamento. Esses achados são resumidos na Tabela 2 .

### Recidiva

Não houve recidiva em pacientes de nenhum dos grupos ( *p* = 1), como mostra a Tabela 2 .

## Discussão

O TCG do fêmur proximal não foi discutido em profundidade na literatura científica. Conforme nossa busca bibliográfica, este estudo é o primeiro a enfocar o papel do denosumabe no TCG do fêmur proximal. A maioria dos artigos já publicados se concentrou nos diferentes tipos de reconstruções após a ressecção do TCG do fêmur proximal. 7 Nos concentramos no TCG de grau III do fêmur proximal, excluindo os casos de TCG de graus I e II. O delineamento experimental foi de um estudo comparativo dos resultados com e sem denosumabe neoadjuvante. Também utilizamos denosumabe em dose significativamente menor no pré-operatório, pois estudos recentes utilizaram doses maiores, administradas por mais tempo, para diminuição do estadiamento do tumor ( *downstaging* ), com doses de ataques nos dias 0, 8, 15 e depois uma vez por mês ou a cada 15 dias. 10

A diminuição do estadiamento cirúrgico do TCG com o neoadjuvante denosumabe e sua correlação com o futuro procedimento escolhido têm sido discutidas na literatura. Rutkowski et al. 11 concluíram que o denosumabe foi bem-sucedido no diminuição do estadiamento cirúrgico e no adiamento da cirurgia em 48% dos participantes. No entanto, a taxa de recidiva foi de 15% enquanto doses mensais de denosumabe foram usadas para tratamento de longo prazo. 11 A utilidade em longo prazo do denosumabe foi proposta como fator de risco para alterações malignas sarcomatosas no TCG. 12 Portanto, o uso em curto prazo do neoadjuvante denosumabe associado à cirurgia é uma modalidade ideal e segura. Porém, a escolha do procedimento cirúrgico ainda é a etapa mais crucial para o tratamento.

Antes, o foco principal era considerar a curetagem intralesional do TCG um procedimento de menor morbididade, mas que levava a maiores taxas de recidiva. 13 Errani et al. 14 concluíram que a curetagem intralesional do fêmur proximal era um fator de risco para a recidiva. Portanto, realizamos a ressecção do fêmur proximal seguida por artroplastia em 16 de 18 pacientes e curetagem intralesional em 2 pacientes. Tentamos a curetagem em três casos, mas, em um candidato, o calcar femoral era irrecuperável; assim, convertemos o procedimento para ressecção e artroplastia com prótese de Wagner. Wijsbek et al. relataram melhor resultado com a curetagem do que com a artroplastia no tratamento do TCG do fêmur proximal. 8 Khan et al. 6 incluíram endopróteses personalizadas para tratamento do TCG do fêmur proximal. Do nosso ponto de vista, o sítio do tumor ainda é um indicador melhor para a seleção entre ressecção ou curetagem, em vez da diminuição do estadiamento após a administração de denosumabe.

Avaliamos os desfechos funcionais e oncológicos às 6 semanas e aos 6 e 12 meses para possível detecção de melhora gradual com o passar do tempo. O HSS é tradicionalmente usado em cirurgia esportiva e artroplastia. 15 De acordo com nossos resultados, o HSS e o escore MSTS continuaram significativamente melhores no grupo com denosumabe durante a fase pós-operatória imediata. No entanto, o HSS mostrou um rápido aumento nas funções do quadril, produzindo resultados similares aos 6 meses de acompanhamento. Todos os pacientes tratados com denosumabe relataram melhora significativa. Segundo Petranova et al., 16 o alívio significativo da dor foi relatado por 53,6% dos pacientes com TCG. Logo, a diminuição da dor pré-operatória levou a uma melhor reabilitação e desfecho funcional da articulação do quadril no grupo tratado com denosumabe em nosso estudo.

Estudos recentes associaram o uso neoadjuvante de denosumabe ao aumento da incidência de recidivas. 17 Entretanto, é importante mencionar que esses estudos que relataram aumento de recidivas utilizaram denosumabe com curetagem. 13 Agarwal et al. 18 propuseram que a recidiva após a administração de denosumabe e a realização de curetagem pode ser prevenida pela curetagem profunda do tumor para obtenção de margens pré-terapêuticas condizentes com as radiografias concomitantes. A borda osteoesclerótica protege certos remanescentes de células gigantes e a curetagem leva à recidiva nos primeiros 2 anos após a cirurgia primária. Nossos resultados não mostraram incidência de recidiva em nenhuma coorte, indicando a ausência de associação entre o denosumabe e a recidiva.

O denosumabe forma uma borda osteoesclerótica ao redor do tumor, o que facilita o manuseio e a ressecção da neoplasia e diminui o tempo intraoperatório, como demonstrado por Sahito et al. 19 e Muller et al. 20 A diminuição do tempo intraoperatório reduz as chances de crescimento microbiano, como demonstrado por Cheng et al. 21 e Teo et al. 22 Assim, em nosso estudo, houve um caso de infecção profunda em uma cirurgia de revisão no grupo sem denosumabe. Além disso, a cirurgia primária foi realizada em até 108 minutos, o tempo intraoperatório mais longo entre todos os 18 casos operados.

## Conclusão

Concluindo, o denosumabe provou ser benéfico no tratamento do TCG do fêmur proximal ao permitir reabilitação precoce e melhorar os desfechos cirúrgicos e oncológicos. No entanto, a diminuição do estadiamento do TCG do fêmur proximal para resgate da articulação do quadril ainda é questionável e requer mais pesquisas. Além disso, os pacientes que não estão dispostos ao tratamento ou com efeitos adversos graves ao denosumabe devem ser aconselhados sobre o progresso e a reabilitação mais lentos e as dificuldades pós-operatórias iniciais, especialmente a dor. Também concluímos que a administração semanal de denosumabe por 4 semanas antes da cirurgia é ideal e que pode não haver necessidade de doses adicionais.

## References

[1] Sahito B Ali S ME Farooqui S F Abro A Ahmed J Younis Resection and reconstruction with and without neoadjuvant denosumab in campanacci grade III giant cell tumors of proximal humerus: a retrospective comparative study Eur J Orthop Surg Traumatol 2023 33 01 81 88 10.1007/s00590-021-03162-2 34773494

[2] Olivera P Perez E Ortega A Terual R Gomes C Moreno L F Estrogen receptor expression in giant cell tumors of the bone Hum Pathol 2002 33 02 165 169 10.1053/hupa.2002.31476 11957140

[3] Campanacci M Baldini N Boriani S Sudanese A Giant-cell tumor of bone J Bone Joint Surg Am 1987 69 01 106 114 3805057

[4] Sahito B Ali S ME Qamar J Katto M S Ahmed M W Jamil M A Comparison of Outcomes of ‘Extensor Carpi Ulnaris Tenodesis’ versus ‘No Tenodesis’ after Resection of the Distal Ulna in Patients with Giant Cell Tumor J Hand Surg Asian Pac Vol 2022 27 01 110 116 10.1142/S2424835522500102 35037577

[5] Rutkowski P Gaston L Borkowska A Stacchiotti S Gelderblom H Baldi G G Denosumab treatment of inoperable or locally advanced giant cell tumor of bone - Multicenter analysis outside clinical trial Eur J Surg Oncol 2018 44 09 1384 1390 10.1016/j.ejso.2018.03.020 29650420

[6] Khan S A Kumar A Inna P Bakhshi S Rastogi S Endoprosthetic replacement for giant cell tumour of the proximal femur J Orthop Surg (Hong Kong) 2009 17 03 280 283 10.1177/230949900901700306 20065363

[7] Gosal G S Boparai A Makkar G S Long-Term Outcome of Endoprosthetic Replacement for Proximal Femur Giant Cell Tumor Niger J Surg 2015 21 02 143 145 26425070 10.4103/1117-6806.162583 PMC4566322

[8] Wijsbek A E Vazquez-Garcia B L Grimer R J Giant cell tumour of the proximal femur: Is joint-sparing management ever successful? Bone Joint J 2014 96-B 01 127 131 10.4103/1117-6806.162583 24395323

[9] Iamthanaporn K Chareancholvanich K Pornrattanamaneewong C Reasons for revision of failed hemiarthroplasty: Are there any differences between unipolar and bipolar? Eur J Orthop Surg Traumatol 2018 28 06 1117 1123 10.1007/s00590-018-2176-0 29549451

[10] Jamshidi K Gharehdaghi M Hajialiloo S S Mirkazemi M Ghaffarzadehgan K Izanloo A Denosumab in Patients with Giant Cell Tumor and Its Recurrence: A Systematic Review Arch Bone Jt Surg 2018 6 04 260 268 30175172 PMC6110426

[11] Rutkowski P Ferrari S Grimer R J Stalley P D Dijkstra S PD Pienkowski A Surgical downstaging in an open-label phase II trial of denosumab in patients with giant cell tumor of bone Ann Surg Oncol 2015 22 09 2860 2868 10.1245/s10434-015-4634-9 26033180 PMC4531146

[12] Alaqaili S I Abduljabbar A M Altaho A J Khan A A Alherabi J A Malignant Sarcomatous Transformation of Benign Giant Cell Tumor of Bone after Treatment with Denosumab Therapy: A Literature Review of Reported Cases Cureus 2018 10 12 e3792 10.7759/cureus.3792 30868006 PMC6402735

[13] Sano K Suehara Y Okubo T Sasa K Kurihara T Akaike K Preoperative denosumab treatment with curettage may be a risk factor for recurrence of giant cell tumor of bone J Orthop Surg (Hong Kong) 2020 28 02 2.309499020929786E15 10.1177/2309499020929786 32539628

[14] Errani C Tsukamoto S Leone G Akahane M Cevolani L Tanzi P Higher local recurrence rates after intralesional surgery for giant cell tumor of the proximal femur compared to other sites Eur J Orthop Surg Traumatol 2017 27 06 813 819 10.1007/s00590-017-1983-z 28589498

[15] Lau B C Scribani M Lassiter T Wittstein J Correlation of Single Assessment Numerical Evaluation Score for Sport and Activities of Daily Living to Modified Harris Hip Score and Hip Outcome Score in Patients Undergoing Arthroscopic Hip Surgery Am J Sports Med 2019 47 11 2646 2650 10.1177/0363546519863411 31348867

[16] Petranova T Sheytanov I Monov S Nestorova R Rashkov R Denosumab improves bone mineral density and microarchitecture and reduces bone pain in women with osteoporosis with and without glucocorticoid treatment Biotechnol Biotechnol Equip 2014 28 06 1127 1137 10.1080/13102818.2014.967827 26019600 PMC4434053

[17] Chen X Li H Zhu S Wang Y Qian W Pre-operative denosumab is associated with higher risk of local recurrence in giant cell tumor of bone: a systematic review and meta-analysis BMC Musculoskelet Disord 2020 21 01 256 10.1186/s12891-020-03294-2 32312263 PMC7171828

[18] Agarwal M G Gundavda M K Gupta R Reddy R Does Denosumab Change the Giant Cell Tumor Treatment Strategy? Lessons Learned From Early Experience Clin Orthop Relat Res 2018 476 09 1773 1782 10.1007/s11999.0000000000000243 30794215 PMC6259809

[19] Sahito B Ali S ME Kumar D Kumar J Hussain N Lakho T Role of denosumab before resection and reconstruction in giant cell tumors of bone: a single-centered retrospective cohort study Eur J Orthop Surg Traumatol 2022 32 03 567 574 10.1007/s00590-021-03012-1 34050817

[20] Müller D A Beltrami G Scoccianti G Campanacci D A Franchi A Capanna R Risks and benefits of combining denosumab and surgery in giant cell tumor of bone-a case series World J Surg Oncol 2016 14 01 281 10.1186/s12957-016-1034-y 27809843 PMC5095954

[21] Cheng H Chen B P Soleas I M Ferko N C Cameron C G Hinoul P Prolonged Operative Duration Increases Risk of Surgical Site Infections: A Systematic Review Surg Infect (Larchmt) 2017 18 06 722 735 10.1089/sur.2017.089 28832271 PMC5685201

[22] Teo B JX Yeo W Chong H C Tan A HC Surgical site infection after primary total knee arthroplasty is associated with a longer duration of surgery J Orthop Surg (Hong Kong) 2018 26 02 2.309499018785647E15 10.1177/2309499018785647 30010488

